# Bacterial diversity in rhizosphere of *Paspalum scrobiculatum* L. (kodo millet) is revealed with shotgun metagenome sequencing and data analysis

**DOI:** 10.1016/j.dib.2018.09.006

**Published:** 2018-09-07

**Authors:** Ratna Prabha, Dhananjaya P. Singh, Mukesh K. Verma, Pramod Sahu, Prafull Kumar

**Affiliations:** a Chhattisgarh Swami Vivekananda Technical University, Bhilai, Chhattisgarh 491107, India; b ICAR-National Bureau of Agriculturally Important Microorganisms, Indian Council of Agricultural Research, Kushmaur, Maunath Bhanjan 275101, UP, India; c S. G. College of Agriculture and Research Station, Indira Gandhi Krishi Vishwavidyalaya, Raipur, Jagdalpur, Chhatisgarh 494001, India

## Abstract

Rhizosphere bacterial communities of kodo millet plant was analyzed from a large metagenome sequence dataset. Plant rhizosphere samples of kodo millet was collected in replicates and the metagenomic sequence data were obtained through shotgun sequencing. Overall sequences in the dataset were 476,649 comprising total read length of 179,349,372 base pairs. Taxonomic data analysis led to characterize α-diversity of 107 species. Dominance of actinobacteria followed by unclassified sequences (derived from Bacteria) was recorded. Raw data along with the analysis result is publicly available from the MG-RAST server with ID mgm4761530.3.

**Specification table**

**Table:** 

Subject area	*Biology*
More specific subject area	*Metagenomics*
Type of data	*DNA sequences*
How data was acquired	Shotgun DNA sequencing using Illumina HiSeq
Data format	Analyzed data
Experimental factors	Collection of rhizosphere in replicates, extraction of metagenomic DNA from the rhizosphere of 2 months old kodo plants
Experimental features	Shotgun sequencing of the metagenomic DNA followed by bioinformatics analysis for microbial community composition
Data source location	Jagdalpur, Chhattisgarh, India (latitude: 19.07 and longitude: 81.96)
Data accessibility	Data is available from MG-RAST server (ID: mgm4761530.3) (http://metagenomics.anl.gov/mgmain.html?mgpage=overview&metagenome=mgm4761530.3).
Related research article	None

**Value of the data**

• The data highlights rhizosphere bacterial diversity of kodo millet plants grown under low-fertility soils and drought-prone conditions.
• Analysis reveals dominance of actinobacteria in the rhizosphere of kodo plant.
• The dataset shows diversity of plant growth promoting bacteria (PGPB).
• The data enhances our understanding on dominant microbial inhabitants of millet rhizosphere that may further be exploited for growing crops under harsh abiotic conditions and low-fertility soil status.

## 1 Data

**Fig. 1:**
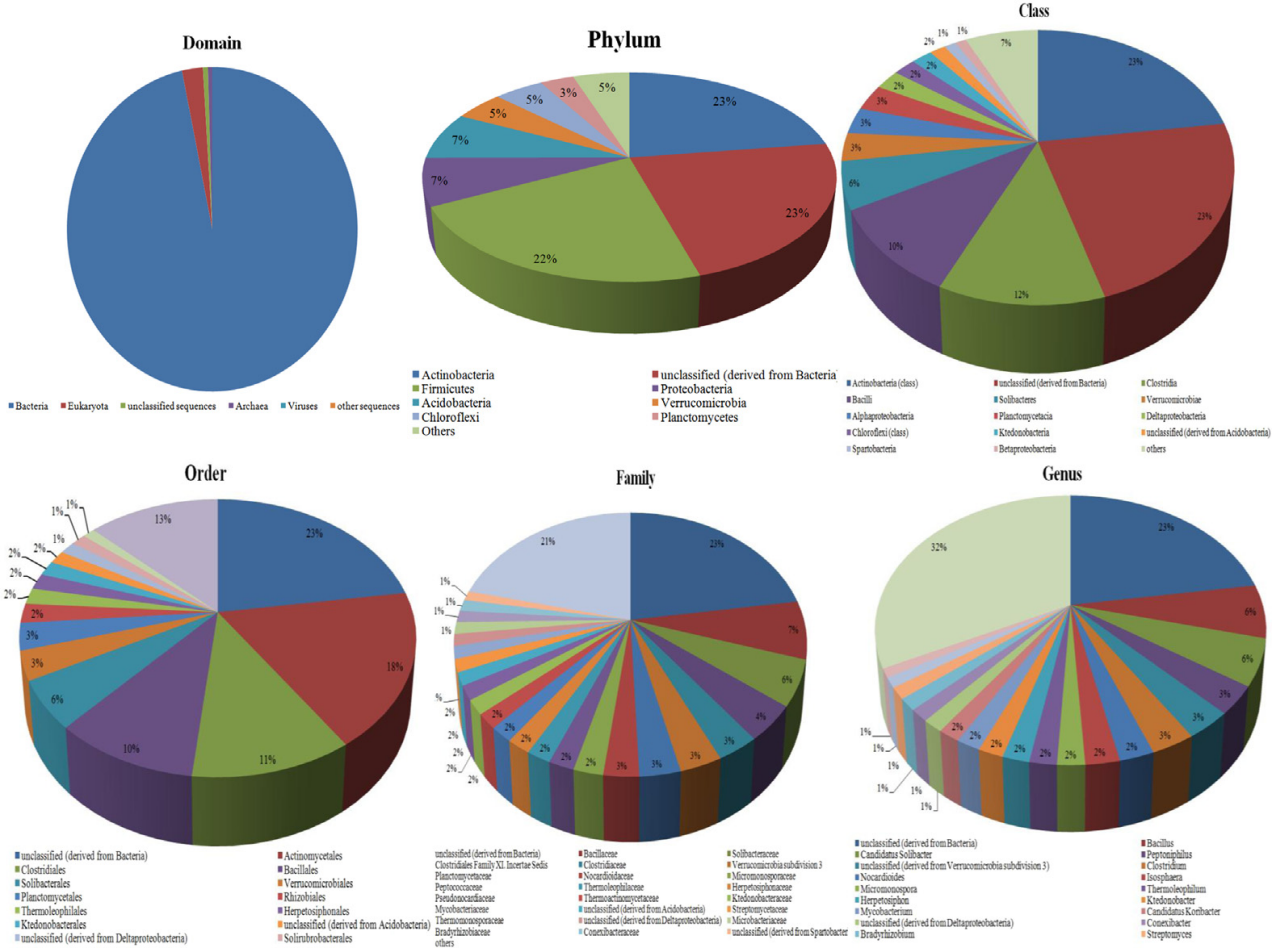
Abundance of bacterial communities at different taxonomic units (Domain, Phylum, Class, Order, Family and Genus). Groups occupying less than 1% of the distribution were clubbed together and was designated as ׳Others׳.

**Fig. 2:**
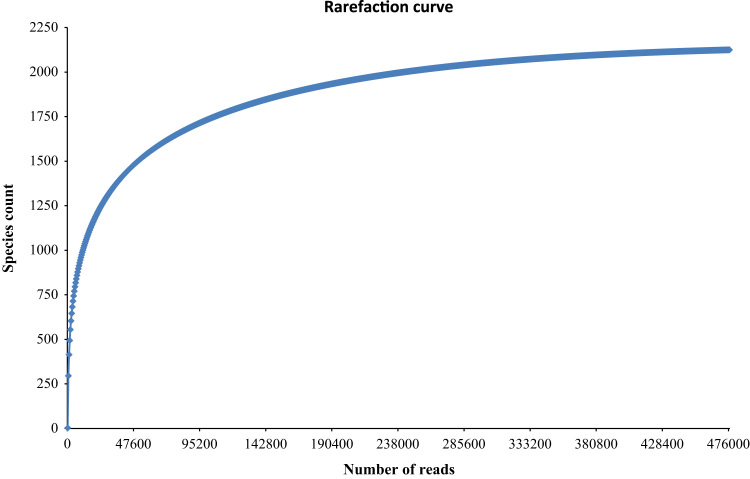
Rarefaction curve of species richness.

**Fig. 3:**
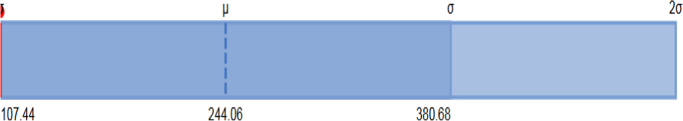
α-diversity of the data set. The min, max, and mean values along with the standard deviation ranges (σ and 2σ) in varying shades are shown. The α-diversity of this metagenome dataset is shown in red. Alpha diversity sum up the diversity of organisms in a particular sample by a single number.

The rhizosphere metagenomic shotgun sequencing data was obtained. Total number of sequences were 476,649 with total read length of 179,349,372 base pairs (Table 1). Bacterial community structure in the kodo rhizosphere is reflected in Fig. 1, species richness in Fig. 2 and the α-diversity of 107 species is shown in Fig. 3.

**Table 1:** Details about the raw and processed sequences of the kodo rhizosphere metagenome.

Information about uploaded data	
Number of basepair	179,349,372 bp
Number of sequences	476,649
Mean sequence length	376 ± 76 bp
Mean GC percent	57 ± 3%
Information after quality control analysis	
bp count	22,138,479 bp
Sequences count	98,133
Mean sequence length	226 ± 124 bp
Mean GC percent	57 ± 3%
About processed sequences	
Predicted protein features	679
Predicted rRNA features	34,247

Out of total reads, 96.66% were assigned to bacteria (Supplementary Table 1). Actinobacteria was the most dominant phylum (22.76%) followed by unclassified bacteria (22.64%) and Firmicutes (22.2%) (Supplementary Table 2). Dominance of actinobacteria was also evident at the class level (Supplementary Table 3). At the order level, unclassified bacteria and Actinomycetales were the most dominating communities (Supplementary Table 4). Unclassified bacteria were also observed at family (Supplementary Table 5) and genus (Supplementary Table 6) level, though families of actinobacteria also exhibited significant proportion.

## 2 Experimental design, materials, and methods

### 2.1 Sample collection

Rhizosphere samples of kodo plants was obtained from the field of the College of Agriculture, Jagdalpur, Chhattisgarh, India (19.07N;81.96E) in April 2017.

### 2.2 DNA extraction

Total DNA was isolated through the FastDNA™ SPIN Kit following manufacturer instructions. Community DNA was purified and characterized through agarose-gel electrophoresis and NanoDrop spectrophotometer.

### 2.3 Metagenome sequencing

For the isolated DNA, amplicon sequencing was carried out with Illumina HiSeq sequencing system.

### 2.4 Initial pre-processing and QC check

The paired end fastq read files of the rhizosphere metagenomic dataset was processed through the standard pipeline of MG-RAST server [1] with default parameters.

### 2.5 Taxonomic analysis

For the taxonomic assignments, dataset was processed via MG-RAST server [1] by aligning the reads against the RefSeq protein database which provides search against various sequence databases at the same time [1]. Parameters taken were maximum *E*-value: 1 × 10^−5^, minimum percentage identity: 60%, and minimum alignment length: 15.
