# Supplementary material for: Bacterial diversity in rhizosphere of Paspalum scrobiculatum L. (kodo millet) is revealed with shotgun metagenome sequencing and data analysis
Source: Data Brief. 2018 Sep 7;20:1653–7. doi: 10.1016/j.dib.2018.09.006 (PMC6157409; doi:10.1016/j.dib.2018.09.006)
Supplement: Supplementary file 1 — Supplementary material [file mmc1.docx]

Conflict of Interest

Authors declared that there exists no conflict of interest.
